# Advances in the Molecular Regulatory Mechanisms of Testicular Development and Spermatogenesis in Yaks

**DOI:** 10.3390/ani16172730

**Published:** 2026-09-02

**Authors:** Qiqi Yin, Xinxing Zheng, Xingdong Wang, Yongming Zhang

**Affiliations:** 1 College of Life Science and Technology, Inner Mongolia Normal University, Hohhot 010022, China; yqq011124@163.com (Q.Y.);; 2 Key Laboratory of Biodiversity Conservation and Sustainable Utilization in Mongolian Plateau for College and University of Inner Mongolia Autonomous Region, Hohhot 010022, China

**Keywords:** testis development, spermatogenesis, cattle-yak, hybrid sterility, high-altitude adaptation, single-cell transcriptomics

## Abstract

Yaks are indigenous livestock species of the Qinghai–Tibet Plateau. Due to long-term exposure to extreme environmental stressors, including high altitude, hypoxia, and low temperatures, its male reproductive system has evolved distinct adaptive strategies. Specifically, testicular weight is markedly lower than that of cattle. Nevertheless, according to reported data from different experiments, although its single ejaculate volume is lower than that of Tibetan cattle, it can still maintain comparable fresh sperm motility. These reproductive phenotypes make the yak an ideal model organism for investigating plateau adaptation and sperm energy metabolism. The present review synthesizes continuous molecular events in yak testes spanning embryonic development through senescence. It focuses on dissecting the core regulatory networks governing spermatogonial stem cell self-renewal, meiosis, and spermiogenesis. It integrates the latest advances in testicular microenvironment dynamics and epigenetic modifications. Additionally, male sterility in cattle-yak serves as a natural mutant model. Essential regulatory modules governing spermatogenesis can be inferred from its spermatogenic-arrest phenotype. Nevertheless, comparative omics analyses between yaks and cattle are confounded by seasonal variation and differing genetic backgrounds. It is therefore critical to distinguish signatures driven by genetic adaptation from those arising from environmental plasticity. Finally, this paper presents research prospects regarding how to utilize single-cell multi-omics and gene-editing technologies to deeply dissect the underlying mechanisms of plateau reproductive adaptation to provide theoretical support for improving yak fecundity and hybrid breeding.

## Simple Summary

The yak is a species endemic to the Qinghai–Tibetan Plateau and exhibits notable male reproductive traits under hypoxic and low-temperature stress. Specifically, testicular weight is markedly lower than that of cattle. Nevertheless, according to reported data from different experiments, although its single ejaculate volume is lower than that of Tibetan cattle, it can still maintain comparable fresh sperm motility. These features make it an ideal natural model for investigating the interplay between energy metabolism and reproductive adaptation. This review summarizes molecular regulatory dynamics throughout the full-life-cycle development of yak testes, highlighting core molecular networks governing spermatogonial stem-cell self-renewal, meiotic initiation, and sperm morphogenesis. It further integrates the modulatory roles of the testicular microenvironment and epigenetic modifiers into a unified conceptual framework. By taking advantage of male sterility in cattle-yak, essential checkpoints governing spermatogenesis can be inferred from its defective phenotype. Finally, we discuss prospects for leveraging single-cell multi-omics and gene-editing technologies to deepen mechanistic understanding of high-altitude reproductive adaptation. These insights may provide theoretical underpinnings for improving yak fertility and supporting crossbreeding programs.

## 1. Introduction

The testis is a highly specialized male reproductive organ in mammals, and its development and function are influenced by intrinsic factors and the external environment. Mechanisms governing testicular development and spermatogenesis differ substantially across species [1]. As a livestock species endemic to the Qinghai–Tibet Plateau, yaks display notable adaptive reproductive biological characteristics under high-altitude environments [2]. Such adaptability not only influences the economic development of local livestock farming, but is also for sustaining plateau ecosystem stability and preserving agricultural biodiversity [3]. Such adaptations are specifically reflected in seasonal reproduction, delayed sexual maturity, and comparatively low reproductive efficiency [3]. As critical determinants of male reproductive performance, testicular development and spermatogenesis in yaks have attracted growing research interest. Enabled by high-throughput approaches including transcriptomics, non-coding RNA profiling, and epigenetics, researchers are progressively unraveling the molecular mechanisms governing reproductive regulation in yaks. This paper reviews the key regulatory factors influencing yak testicular development and spermatogenesis, with a particular focus on the molecular features associated with high-altitude adaptation, and explores potential future research directions in this field.

This review employs cattle-yak male sterility as a genetic comparative model. As F_1_ interspecific hybrids derived from taurine cattle sires and yak dams, cattle-yak males exhibit sterility consistent with Haldane’s rule. This sterility originates from genetic incompatibilities triggered by interspecific hybridization between cattle and yaks, rather than representing an adaptive phenotype driven by plateau-specific environmental pressures. Analysis of spermatogenic arrest in cattle-yaks facilitates identification of core spermatogenesis-essential genes. Nevertheless, findings from cattle-yak studies illuminate only fundamental spermatogenic regulation and cannot be directly interpreted as evidence for yak reproductive adaptation to high-altitude habitats.

## 2. Method

To sort out the molecular regulatory mechanisms of yak testicular development and spermatogenesis, we conducted a literature search using PubMed, Web of Science and CNKI databases. The retrieval time span was mainly from 2000 to July 2026, covering major research advances over the past two decades and including selected early-stage classic literature. The included literature covered two research themes: one focused on testicular development and high-altitude reproductive adaptation mechanisms in purebred yaks; the other comprised studies related to male sterility in cattle-yak hybrids, which served as a genetic tool model for inferring the essential molecular events of spermatogenesis. The key search terms used in each combination included but were not limited to “yak”, “cattle-yak”, “testis”, “testicular development”, “spermatogenesis”, “spermatogonial stem cells”, “meiosis”, “spermiogenesis”, “molecular regulation”, “gene expression”, “single-cell transcriptomics”, “epigenetics”, and “non-coding RNA”. Boolean logical operators (AND) were employed for term concatenation, with adjustments made according to the syntax rules of each database.

**Figure 1:**
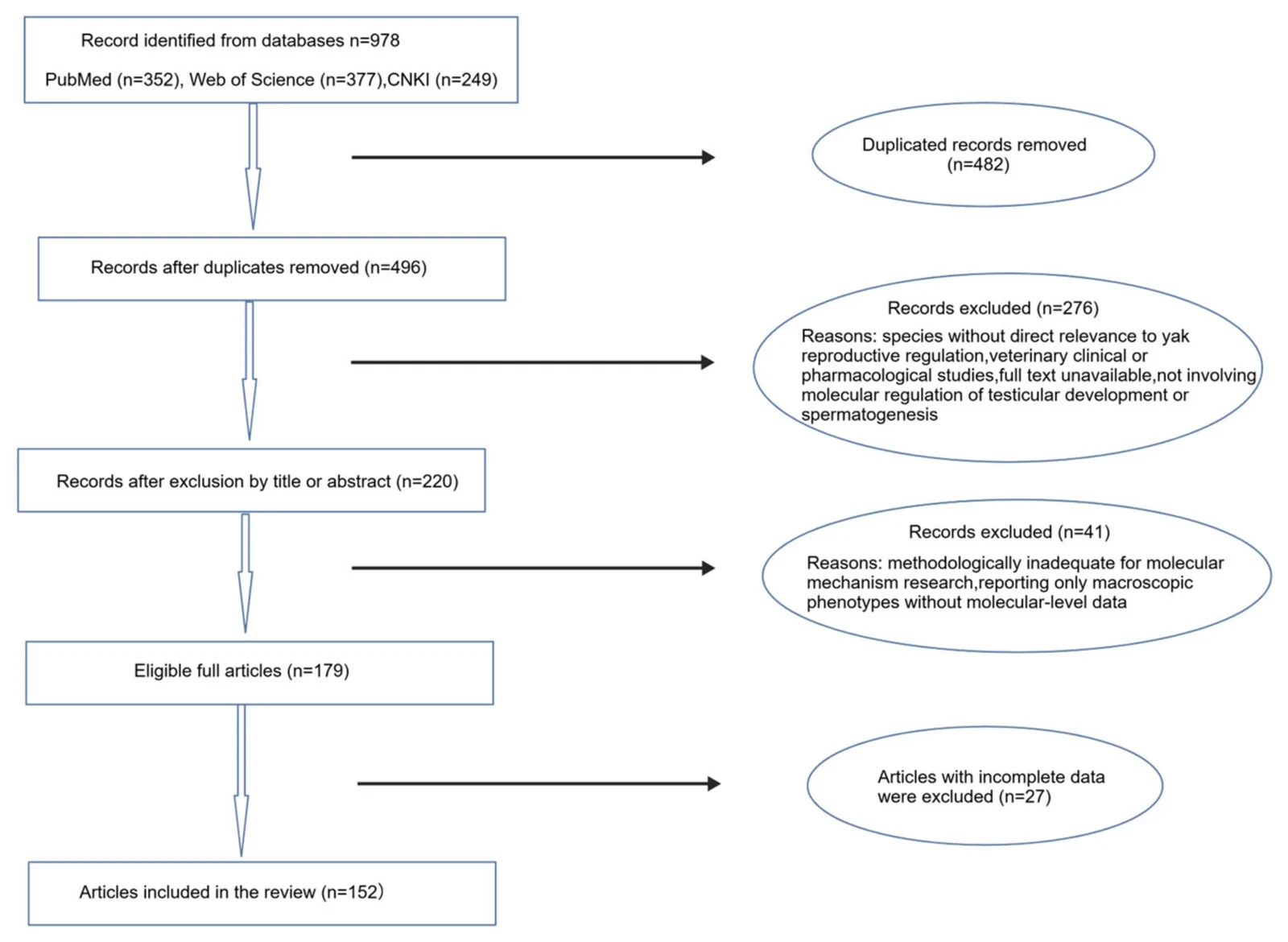
PRISMA-style flow diagram illustrating the literature screening and selection procedure for this review.

A total of 978 records were retrieved from databases, and 152 of these were ultimately included to compile this review. The selection process was summarized in a PRISMA-style flow diagram (Figure 1).

## 3. Biological Basis of Yak Testicular Development

Testicular development plays a pivotal role in yak reproductive system formation and is characterized by a distinct developmental trajectory and regulatory profile. Under the unique high-altitude environment, the postnatal testis of yaks exhibits a developmental pattern distinct from that of plain-dwelling cattle breeds, beginning as early as the embryonic stage. However, relevant research data on the embryonic phase of this process remain relatively scarce. Developmental timing also differs: the period for yaks to reach sexual maturity is longer than that of ordinary cattle. This phenomenon may reflect an evolutionary trade-off in energy allocation and reproductive development in high-altitude species. Through comparative morphological and transcriptomic analyses, researchers found that the temporal sequence of multiple key regulatory nodes during yak testis development was altered [4,5]. This feature is hypothesized to correlate with yaks’ adaptation to extreme high-altitude environments, although this awaits further experimental validation.

### 3.1. Embryonic Gonadal Differentiation

Embryonic gonadal differentiation in yaks is the initial reproductive development stage, governed by a complex molecular network and may differ from low-altitude cattle. The primordial gonad of mammalian testes originates from the genital ridge formed by the intermediate mesoderm during embryonic development and develops via co-differentiation of the coelomic epithelium and mesonephric mesenchyme [6,7,8]. Mammalian embryonic testicular development proceeds through sequential morphological events: genital-ridge elevation, primitive sex-cord formation, testicular-cord differentiation, tunica albuginea establishment, and final organ morphogenesis [9]. After birth, the seminiferous tubules remain immature until puberty, when they achieve functional maturation under hormonal regulation [10]. This process is a complex biological event involving the temporally coordinated regulation of multiple genes [11]. Owing to the extreme scarcity of yak embryonic sample data, most inferences regarding gonadal differentiation in yak embryos are derived from conserved mechanisms in other mammals.

In mammalian sex determination, the master gene *SRY* is activated first to induce the expression of downstream *SOX9*; together, they drive the differentiation of the genital ridge toward testis formation [7,12,13]. Although gonadal developmental gene expression profiles have been reported using cattle as the model [14] direct experimental evidence regarding the temporal expression pattern of the *SRY/SOX9* pathway in yak embryos is still lacking, as yaks are a bovine species endemic to high-altitude regions. Whether there are temporal expression differences in this pathway between yaks and cattle and whether such potential differences are involved in high-altitude adaptive evolution remain to be experimentally verified using embryonic gonadal samples from yaks. During the early stages of gonadal differentiation, *SOX9* acts as a direct downstream effector of *SRY*, directing the lineage commitment of Sertoli cells (SCs), and its expression level is closely associated with sex cord formation. In mouse models, *NR2F2*, *HGF*, and its receptor c-Met regulate the differentiation and physiological functions of embryonic Leydig cells (LCs) [15,16]. Genes including *SOX9*, *GATA4*, and anti-Müllerian hormone (*AMH*) occupy central roles in SC differentiation and Müllerian duct regression [17]. Direct experimental evidence for the functions of the above-mentioned genes in yak embryonic gonads is still lacking, and their roles are only speculated based on conserved mechanisms in other mammals.

The migration of primordial germ cells (PGCs) plays a pivotal role in the differentiation of the embryonic gonads. In model mammals, PGCs migrate from the yolk sac wall to the genital ridge. This process is precisely regulated by the chemokine *CXCL12* (*SDF-1*) and its receptor *CXCR4*, and the integrity of migration directly determines subsequent gonadal morphogenesis. In addition, *CXCL12*, stromal transcription factors *NR2F2* and *TCF21*, together with the peritubular cell marker *ACTA2*, cooperatively participate in germ cell migration and gonadal microenvironment establishment [16,18,19]. After PGCs successfully colonize the genital ridges, they interact with somatic cells to form primitive sex cord structures, at which point the supporting cells begin to secrete *AMH*. *AMH* not only regulates the regression of the Müllerian ducts but also participates in the formation of the rete testis and the regulation of LC differentiation. Studies in model animals have confirmed that, in the maintenance of germ-cell identity, *NANOG* ensures the survival of PGCs [20], *DDX4* regulates their proliferation [21], and *TCFAP2C* suppresses the somatic differentiation program [22]. Currently, there is no direct experimental verification of the functions of the above-mentioned molecules in yak embryonic PGCs.

The establishment of sex cord structure is an important marker of testicular development. The developmental timing of embryonic sex cords in yaks may be delayed relative to plain cattle. This morphological hypothesis and its potential intrinsic association with high-altitude adaptation remain to be experimentally validated. In embryonic testes, supporting cell precursors form tubular structures through intercellular junctions, enclosing PGCs to constitute the rudiment of the seminiferous tubules. LC differentiation and angiogenesis proceed synchronously, jointly generating the nutritional supply and hormonal microenvironment required for testicular development. In steroidogenesis, *STAR* is the rate-limiting protein for cholesterol transport [23], and in humans, *HSD3B2* and *CYP17A1* synergistically participate in androgen synthesis [24,25]. The activation characteristics of this pathway in yak embryonic testes remain to be elucidated.

Epigenetic regulators are equally indispensable for gonadal differentiation during yak embryogenesis. *PIWIL4* (*MIWI2*) mediates transposon silencing and genomic methylation in mice and other models [26,27]. Subsequent experiments showed that its expression in the yak testis increases significantly with sexual maturation, and this upregulated expression confirms that the pathway is conserved in yaks [28]. However, the epigenetic regulator *MSL3* exhibits species differences: it is specifically expressed in primates [18], whereas its knockout in rodents does not affect spermatogenesis [29].

Studies on molecular regulatory networks, together with transcriptomic data, reveal significant differences in gene expression profiles at the key nodes of gonadal differentiation between yaks and low-altitude cattle breeds. These interspecific differences are presumed to affect pathway activity. For instance, functional experiments have verified that inhibition of the WNT/β-Catenin pathway attenuates the proliferative capacity of yak DP cells, whereas activation of this pathway enhances the proliferation of cattle DP cells, indicating substantial inter-species differences in the activation pattern of this pathway between the two species [30]. This is considered an evolutionary strategy for yaks to cope with high-altitude reproductive stress; nevertheless, direct experimental evidence at the yak embryonic level is still lacking to support this hypothesis. Overall, epigenetic regulatory mechanisms of gonadal differentiation during yak embryogenesis remain poorly understood. Further investigation is needed to characterize dynamic changes in DNA methylation and histone modifications [31]. The temporal expression of the above genes collectively drives sex differentiation and tissue organization of the embryonic testis. The completion of gonadal differentiation during the embryonic period establishes the basic structural blueprint of the testis, while the early juvenile stage marks the phase of refinement of the tissue architecture.

Integrating mammalian gonadal-development mechanisms and existing yak transcriptomic datasets, Table 1 summarizes key functional genes participating in embryonic gonadal differentiation, primordial-germ-cell migration and homeostasis, steroidogenesis, and epigenetic regulation. This table compiles their biological functions, target-cell types, experimental-evidence-source species, corresponding developmental stages, and supporting references.

### 3.2. Preparation Stage of the Juvenile Stage

The juvenile phase of yaks is broadly defined as the period spanning from birth to the initiation of sexual maturity. Proteomic and histological evidence shows that the testes of yaks reared at high altitudes have not entered meiosis at six months of age [32]. During this stage, the testis is primarily regulated by focal adhesion and ECM-receptor interaction signaling pathways. Core hub genes such as *SLMAP* participate in microtubule cytoskeleton assembly and coordinate with transcription factors including *GATA* and *WT1*, as well as lncRNAs, to accomplish the establishment of the testis cord architecture [33]. (These results originate from an unpublished doctoral dissertation; relevant observations require further independent experimental validation and formal peer-reviewed publication.) At this stage, the core task is the establishment of the seminiferous tubule scaffold structure, and there remains a long developmental interval before the cytological initiation of spermatogenesis. Transcriptional remodeling is prominent during this developmental window. Many differentially expressed mRNAs, lncRNAs, and circRNAs participate in gonadal mesoderm development and the regulation of spermatogenesis [34]. Specifically expressed circRNAs and miRNAs form an intricate regulatory network via the Wnt, MAPK, and GnRH signaling pathways [5]. At the protein level, the COL1A2 protein exhibits temporal expression changes [32]; ribosome profiling studies indicate that meiotic key genes such as *MEIOB* are primarily regulated by translation efficiency [35], suggesting that the regulatory focus gradually shifts to the translational level during later developmental stages.

Epigenetic modifications are also highly active during early life stages. N^6^-methyladenosine (m^6^A) RNA-methylation profiles differ markedly between juvenile and sexually mature yak testes. This epigenetic mark modulates genes such as *BRCA1* and *STAT3* via Wnt and PI3K-mTOR pathways, governing cell proliferation and maintaining endocrine homeostasis [36]. Small non-coding RNAs also exert critical regulatory effects. A novel abundant small RNA termed mse-tsRNA has been identified in yak sperm, alongside reproduction-associated differentially expressed miRNAs such as miR-19a and miR-142 [37]. At the level of key functional genes, the expression levels of *LYZL4* and *LYZL6* were lowest at 6 months of age and showed a gradual upward trend with increasing age [38,39]; *MMP-2* and *TIMP-2* were mainly localized in germ cells (GCs) and SCs during the neonatal and juvenile stages [40]. The sperm–egg-fusion mediator *IZUMO1* exhibits extremely low transcript abundance before puberty and undergoes pronounced upregulation upon sexual maturation [41]. Therefore, the core task of the yak testis during the juvenile period is to complete the construction of the seminiferous tubule scaffold and cell colonization, thereby providing a structural foundation for subsequent functional maturation. After the seminiferous tubule framework is established during early development, testicular development enters the pubertal functional activation phase as the hypothalamic-pituitary-gonadal (HPG) axis gradually activates.

### 3.3. Regulation of Pubertal Development

The neuroendocrine regulation of pubertal development in yaks primarily depends on the functional activation of the HPG axis. Gonadotropin-releasing hormone (GnRH) secreted by hypothalamic arcuate nucleus neurons exhibits a characteristic pulsatile pattern. Its release profile undergoes dynamic changes throughout ontogeny. GnRH, follicle-stimulating hormone (FSH), luteinizing hormone (LH), testosterone, and inhibin serve as the core functional hormones that collectively regulate the entire process of spermatogenesis through multi-level positive and negative feedback mechanisms [42,43]. Although the differential expression of *GRB2* and *PGRMC1* in the reproductive axis of yaks and yellow cattle supports the inference that HPG-axis maturation may occur later in yaks than in plain-dwelling cattle, which is consistent with their delayed sexual-maturation phenotype, direct histological evidence for GnRH neuron development in yaks is still lacking [44,45]. Available physiological evidence suggests that maturation of the HPG axis is generally delayed in yaks compared with plain-dwelling cattle breeds. This observation aligns with the phenotype of delayed sexual maturation. However, direct histological evidence describing GnRH-neuron development in yaks is still missing. Upon receiving GnRH signals, gonadotrophs in the anterior pituitary synchronously secrete FSH and LH. These two hormones then travel through the peripheral circulation to act on testicular tissues, regulating the physiological functions of SCs and LCs, respectively.

Gonadotropin-receptor expression patterns within testicular tissue directly shape pubertal progression. The FSH receptor (*FSHR*) is primarily localized to the basal membrane region of SCs, and its expression dynamics are closely associated with the lumen formation of seminiferous tubules [46]. It has been hypothesized that the transcriptional activation of the FSH receptor in yak SCs may be delayed relative to ordinary cattle, and this phenomenon is speculated to partially account for the retarded development of the seminiferous epithelium; nevertheless, this hypothesis remains unvalidated. The LH receptor (*LHR*) is primarily located on the surface of LC membranes. It regulates the expression of key enzymes in testosterone synthesis (such as *CYP11A1* and *CYP17A1*) by activating the cAMP-PKA signaling pathway. High-altitude hypoxia may alter the responsiveness of interstitial cells to LH signaling through stabilization of hypoxia-inducible factor (HIF-1α) [5,47]. Furthermore, the expression levels of growth hormone (GH) and insulin-like growth factor 1 (*IGF-1*) in the testes of adult yaks are higher than those in prepubertal individuals, and *IGF-1* can regulate SCs’ proliferation through the *CyclinD1* pathway [48,49]. Prolactin (*PRL*), via its receptor (*PRLR*), synergizes with the *LHR*; both are co-expressed in LCs and spermatogenic cells, and their expression increases with sexual maturation [48,50]. Clock genes also participate in modulating testosterone secretion, thus influencing the developmental timing of the gonads [51].

At the levels of morphogenesis and molecular signaling, testosterone, LH, and FSH collectively drive the development of seminiferous tubules. Circadian-rhythm-related genes cooperate with *NTRK2*, lncRNA *MSTRG.19083.1*, and *miR-429-y* to regulate spermatogenic-cell proliferation and differentiation [32,52,53]. Specifically, lncRNA *MSTRG.19083.1* acts as a ceRNA by sponging *miR-429-y* to upregulate *NTRK2* expression, thereby coordinating the PI3K-Akt and MAPK pathways to drive cell proliferation and maintain microenvironmental homeostasis [52,54,55,56]. The PI3K-Akt pathway is thought to be important for intracellular signal integration, whereas the high-altitude hypoxic environment may be involved in tissue remodeling via HIF-1α signaling [5,47]. However, this association is currently supported mainly by indirect evidence from other species or cellular models, and direct functional and causal evidence in yak tissues is still lacking. At the receptor level, hormone-bound gonadotropin-releasing-hormone receptor (*GnRHR*), *FSHR*, *LHR*, androgen receptor (*AR*), and inhibin β-B subunit (*INHβB*) mediate stepwise negative-feedback signaling throughout the HPG axis [57]. *FSHR* and *LHR* (gonadotropin receptors), together with the inhibin β-B subunit *INHβB*, are specifically expressed in testicular somatic cells and GCs, mediating testosterone synthesis and spermatogenesis [46,48,58,59]. Activated AR complexes positively induce the expression of the *CLDN* family, thereby regulating blood-testis barrier (BTB) homeostasis [60,61]. Thus, puberty represents a pivotal transition in yak testes from structural maturation to functional initiation, after which adulthood is characterized by the maintenance of functional homeostasis.

### 3.4. Adulthood

Adulthood is a critical stage for maintaining testicular functional homeostasis in yaks, involving the coordinated interaction between the structure of the BTB and the spermatogenic epithelium cycle. At this stage, the tight junctions between SCs serve as the core foundation for the integrity of the BTB [62]; LCs stably express ET-1 and eNOS, synergistically regulating microcirculation and testosterone synthesis [63]. At 12 months of age, spermatids (SPTs) begin to differentiate in high-altitude yaks, and the first wave of spermatozoa formation marks the cytological onset of spermatogenesis [64]. Other studies have revealed that LDH-X exhibits no enzymatic activity in 18-month-old yaks but detectable activity at 24 months of age. At this age, the volume density of seminiferous tubules (0.786 mm^3^/mm^3^) and the height of the seminiferous epithelium (85.66 μm) in yaks are close to those of ordinary adult bulls [64]. It can thus be inferred that yak testicular tissue achieves molecular-physiological functional maturation with the complete establishment of the seminiferous epithelium at 24 months of age. Yaks face evolutionary trade-offs in energy allocation under the high-altitude hypoxic conditions of the Qinghai–Tibet Plateau. Consequently, individuals need a prolonged developmental period to achieve stable HPG-axis function, satisfactory body condition, and consistent semen quality. Under practical production conditions, yaks reach sexual maturity and possess strong breeding capacity at 3–5 years of age, while their testicular structure and function gradually decline after 9 years of age [65,66].

The maintenance of testicular function in adulthood relies primarily on post-transcriptional and translational regulation. Full-length transcriptome analysis revealed that core hub genes in adulthood, such as *novel11503*, are enriched in pathways like glycolysis and aldosterone synthesis, suggesting that adulthood places a greater emphasis on maintaining metabolic homeostasis [67]. Single-cell transcriptomic analysis showed that SCs differentiate along a specific trajectory into four consecutive states: Progenitor, Immature-1, Immature-2, and Mature, with the Mature state mainly participating in processes such as apoptosis, protein C-terminal binding, and protein homodimerization activity [33]. (This four-state classification of Sertoli-cell differentiation is derived from the group’s unpublished doctoral thesis; this cell-state model has not yet undergone formal peer review and awaits independent replication.) Guo et al. [68] further demonstrated that adult gene expression is primarily regulated at the translational level, with the expression of key genes such as *STAT1*, *ITGB5*, and *ERBB2* being modulated by sequence features like uORF length and GC content. In terms of homeostasis maintenance, the adult testis preserves tissue homeostasis through the synergistic action of multiple functional proteins: HSP70 and HSP27 counteract stress to maintain the seminiferous epithelium [69]. (Data from an unpublished dissertation without formal peer review.) *AQP1* and *AQP11* cooperatively regulate fluid transport and osmotic balance [70,71]. *GRB10* and *GRB14* sustain endocrine and structural stability [72].

### 3.5. Senescence Period

Significant regressive morphological changes occur in the testes of yaks during the senescence period (9–13 years of age), which provides the direct structural basis for age-related reproductive functional decline [65]. Under light microscopy, marked proliferation of collagen fibers and reticular fibers is observed in the testicular capsule and interstitial tissue; meanwhile, the seminiferous tubules exhibit varying degrees of degeneration with widened intercellular spaces, and some spermatogenic cells slough off [65]. SCs exhibit sparse cytoplasm, irregular nuclear contours, and dilated and vesicular endoplasmic reticulum, as well as an increased number and enlarged volume of secondary lysosomes; in addition, the integrity of the BTB is disrupted owing to the loss of ectoplasmic specializations, with abnormal connections to the basement membrane, and these alterations collectively lead to declined supportive functions [65]. LCs show markedly increased lipid droplets in the cytoplasm; interstitial capillary endothelial cells display tight junctions. Meanwhile, enhanced positive staining for PAS and AB-PAS suggests abnormal glycoprotein deposition [65,73]. The protein levels of MMP-2 and TIMP-2 are elevated again in aged yaks, and their mRNA expression is higher than that in adult yaks (*p* < 0.05) [40]. Therefore, morphological and stromal changes jointly disrupt the homeostasis of the spermatogenic microenvironment, leading to a marked decline in the breeding capacity of aged yaks.

**Figure 2:**
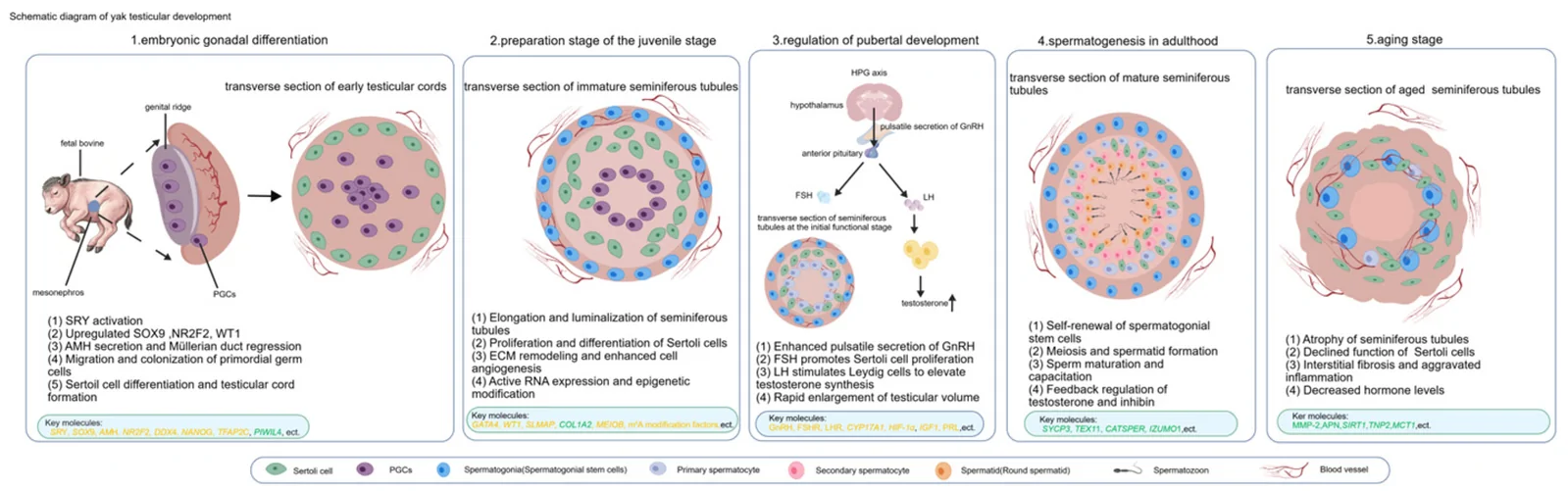
Schematic overview of yak testicular development across the entire lifespan. This diagram summarizes the five successive stages of yak testicular development, including embryonic gonadal differentiation, juvenile preparation, pubertal regulation, adult spermatogenesis, and aging degeneration. For each stage, the corresponding hallmark morphological events and representative molecular regulators are indicated. In this figure, green-colored gene names indicate molecules with direct experimental evidence (expression, protein abundance, or functional validation) in yak tissues; yellow-colored gene names indicate molecules inferred from conserved mechanisms in other mammalian models (mouse, human, cattle) without direct experimental validation in yaks.

At the molecular level, the senescent testis exhibits dysregulation of gene expression networks. Transcriptional levels of adiponectin and its receptors change with age; the mRNA levels of *APN* and *APNR1* reach the highest values in the aged group (*p* < 0.01), and *APNR2* is higher in the juvenile and aged groups than in the lactation and adult groups (*p* < 0.01), whereas the mRNA of the lactate transporter *MCT1* is also elevated in the adult and aged groups (*p* < 0.01), suggesting that senescent cells up-regulate metabolism related transcripts in response to oxidative stress [74]. Although the mRNA of the aging marker protein SIRT1 is widely expressed in various cell types, its protein level continuously declines with age [75]. In contrast, the *TNP2* gene exhibits a specific pattern of divergence between mRNA and protein expression: *TNP2* mRNA is higher in the aged group than in other periods (*p* < 0.01), whereas its protein level is extremely low in the aged group compared with the early and late stages of sexual maturity (*p* < 0.01) [76], indicating that abnormalities in post-transcriptional or translational regulation may occur during the aging process. In summary, yak testicular aging results from the synergistic effects of multiple factors at multiple levels, involving structural degeneration and mechanism remodeling, as well as metabolic adaptation and regulatory imbalance. The overall developmental timeline is schematically summarized in Figure 2.

### 3.6. Species-Specific Traits of Testis Development in Yaks

Combined with the developmental characteristics of each stage described above, yak testes exhibit a species-specific feature of “low-mass high-efficiency”. Wang et al. [77] measured that the average weight of a single testis in adult yaks was 95.80 g, versus 278.53 g for cattle; the weight of the yak epididymis and testis is only about one-third that of cattle. Regarding semen quality, semen parameters have been reported in separate experiments for yaks (semen collected from July to October) and Tibetan cattle (semen collected in summer) [78,79]. Based on these asynchronous and unpaired data, the single ejaculate volume of yaks (3–4 mL) is lower than that of Tibetan cattle (4.80 mL), whereas the range of fresh sperm motility largely overlaps between yaks (67–71%) and Tibetan cattle (55.64–70%). These comparative observations require further replicated empirical validation. The specific values are shown in Table 2.

This phenotypic trait may be closely related to its sophisticated thermoregulatory adaptation. Spermatogenesis is highly temperature-sensitive and needs to be maintained at a temperature 2–4 °C lower than the core body temperature. Because of long-term exposure to plateau environmental stress, yak testes have evolved an efficient counter-current heat-exchange system. The spiral arteries in the spermatic cord are surrounded by a dense pampiniform venous plexus; the number of large collecting veins is reduced, and small veins are densely arranged in a “woven-bag-like” pattern [77]. This vascular configuration increases the surface area for heat exchange, effectively lowering the temperature of testicular arterial blood before it reaches the testis, thereby providing a stable low-temperature microenvironment for spermatogenesis. Furthermore, efficient local blood supply and temperature-regulating mechanisms enable testicular tissue per unit mass to maintain high spermatogenic efficiency, achieving high reproductive capacity with a relatively small organ size.

In addition, as a core molecular mechanism for cells to resist temperature stress, heat shock proteins (HSPs) are specifically highly expressed in yak testes. Studies have found that the relative expression level of HSPA2 in yak testes is higher than that in other tissues and organs. Immunohistochemistry has confirmed strong positive expression of the HSPA2 protein in the cytoplasm and nucleus of spermatogenic cells within the seminiferous tubules of testes [80]. This suggests that yaks have not only optimized thermoregulation at the macroscopic vascular structural level but also evolved sophisticated protective mechanisms against temperature fluctuations at the molecular level.

However, the above-mentioned thermoregulation-related phenotypes are still only characterized at the anatomical and partial molecular levels. The in-depth mechanisms, including the fine regulation of the “woven-bag-like” venous configuration by vascular endothelial growth factor and the complete molecular network through which HSPs cooperate with other stress factors in response to temperature stress, remain to be elucidated, which represents a research direction for future comparative reproductive biology.

### 3.7. Comparison of Testicular Reproductive Adaptability Among Yaks, Tibetan Sheep, and Tibetan Pigs

In the context of reproductive adaptability of male mammals on the Tibetan Plateau, the yak and Tibetan sheep exhibit a convergent strategy characterized by “optimized vascular patterning”, while the Tibetan pig follows an alternative route of “earlier developmental chronology”.

From the perspective of testicular morphology, the unilateral testicular weight in yaks represents merely 34% of that in domestic cattle, while the corresponding ratio of total epididymal length is 0.74, which is substantially higher than the testis weight ratio of 0.34, suggesting that the epididymis occupies a larger relative volumetric proportion [77,81]. The testicular weight of Tibetan sheep is 1.18 times that of small-tailed Han sheep, and the ratio of longitudinal to transverse diameter of the caput epididymidis in Tibetan sheep consistently exceeds 1.13. Combined with the characteristic of increased relative epididymal mass observed in yaks, these findings suggest that both yaks and Tibetan sheep may adapt to high-altitude cold environments through compensatory enlargement of the epididymis [81,82,83]. (Some data from an unpublished dissertation without formal peer review.) At the level of vascular architecture, yaks and Tibetan sheep exhibit the typical configuration of spiral and tortuous arteries in the spermatic cord of the testis. The spiral arteries are enveloped by the pampiniform venous plexus to form a counter-current heat-exchange system, and both species present a “centripetal-priority” blood flow distribution pattern. The diameter-ratio values of centripetal arteries and knot-like arteries in Tibetan sheep are both greater than 1 (1.00–1.22), whereas those of centrifugal arteries are less than 1 (0.76–0.92) [81,82]. In addition, the interspecific diameter ratios of the testicular artery in yaks range from 0.56 to 0.91 [81]. This finding indicates that the reduction in testicular arterial diameter in yaks is relatively less pronounced than the decline in testicular weight. Future investigations should employ testicular-mass-normalized vascular cross-sectional area to further quantitatively assess the blood supply per unit of tissue. Although yaks and Tibetan sheep share highly similar overall vascular configurations, subtle differentiations exist relative to cattle. The diameter of the straight segment of yak testicular arteries gradually decreases along the dorsal side, while that in cattle gradually increases, suggesting adaptive differentiation of interspecific vascular regulatory patterns [77,81].

By contrast, Tibetan boars, lacking specialized vascular architecture, rely on extremely early puberty (5–6 months) to cope with the alpine environment. Their testicular weight increases most dramatically (5.81 times) between 3 and 4 months of age, whereas the epididymis attains its maximum growth rate at 6 months of age [84]. Taken together, yaks and Tibetan sheep improve the blood perfusion efficiency per unit testicular mass via elaborate optimization of vascular architecture, while Tibetan pigs attain reproductive compensation by accelerating the onset of sexual maturity. These distinct patterns collectively illustrate the dual influence of species-specific body size and life-history strategies on testicular adaptability to high-altitude environments.

## 4. Regulatory Network of Spermatogenesis

Spermatogenesis is a finely regulated process of proliferation and differentiation of GCs to produce mature spermatozoa [85]. Spermatogenesis normally takes place in the seminiferous tubules after puberty. Spermatogonia residing in the basal compartment of seminiferous tubules proliferate and differentiate via mitosis to form primary spermatocytes (Pri-SPC). Subsequently, primary spermatocytes initiate meiosis and sequentially pass through five substages of prophase I: leptotene, zygotene, pachytene, diplotene, and diakinesis [86]. Upon completion of meiosis I, Pri-SPCs give rise to secondary spermatocytes (Sec-SPCs). Sec-SPCs rapidly enter meiosis II to produce SPTs. The SPTs undergo complex morphological transformation and ultimately develop into mature spermatozoa [87,88].

### 4.1. Spermatogonial Renewal and Differentiation

The proliferation and differentiation of spermatogonia mark the initiation of spermatogenesis. As the only adult stem cells in the male germline capable of transmitting genetic information to offspring, spermatogonial stem cells (SSCs) play a pivotal role in this process. Yak spermatogenesis originates from type A SSCs, which undergo mitotic self-renewal or differentiate into intermediate-type spermatogonia. These subsequently produce type B_1_ and B_2_ spermatogonia, with type B_2_ ultimately dividing to form primary spermatocytes [89]. Using PAS staining, the cycle of the seminiferous epithelium can be divided into 12 stages, each with a fixed pattern of cell associations. This differentiation time course is largely consistent with that of cattle, demonstrating that the spermatogonial proliferation mode in Bos is highly conserved [90].

At the level of molecular markers and cell identification, the expression pattern of the chemokine receptor *CXCR4* is cell-specific. It is detected in GCs and a subset of spermatogonia, making it an ideal molecular marker for the isolation and identification of yak SSCs [4]. In addition, in the closely related species, ordinary cattle, *GFRα1* has been validated as a specific surface marker for undifferentiated germ cells. *GFRα1*-positive cells account for only 0.6% of the testicular cell population, and *GFRα1*-based flow-cytometric sorting can improve xenotransplantation colony-formation efficiency by 4.4-fold [91]. Although the expression pattern of *GFRα1* in yaks remains to be experimentally verified, these findings provide important references for the combined or comparative application of distinct surface markers in yaks. The transition from PGCs to spermatogonia in yaks exhibited 11,904 significantly differentially expressed transcripts [4]. The magnitude of these transcriptomic changes far exceeds those observed in subsequent developmental stages, suggesting that this transcriptional profile may reflect a yak-specific regulatory mechanism in reproduction. The timing for the transformation from gonocytes to spermatogonia in yaks is largely consistent with that in ordinary cattle, suggesting that the mechanisms underlying SSC formation may be relatively conserved in yaks [4]. At the molecular regulatory level, similar to most mammals, the self-renewal and differentiation of yak SSCs are precisely regulated by microenvironmental factors. Studies have shown that the expression level of glial cell line-derived neurotrophic factor (*GDNF*) secreted by SCs is positively correlated with the proliferative capacity of SSCs [92]. The expression level of *GDNF* in SCs of cattle-yaks is only 29.4% of that in yaks. However, the transcript levels of *GDNF* and its related genes show an upward trend in SSCs [93]. This pattern indicates that the GDNF pathway is essential for sustaining spermatogonial stem-cell homeostasis. The above compartmentalized imbalance of the GDNF pathway in cattle-yaks further confirms the central role of this pathway in spermatogenesis. This abnormality arises from hybrid genomic incompatibility and does not represent a molecular signature of high-altitude adaptation in yaks. In addition, in vitro *GDNF* supplementation experiments have verified that this factor can effectively promote the proliferation of SSCs. Nevertheless, the causal link between reduced *GDNF* expression in cattle-yak SCs and hybrid infertility remains to be verified in vivo.

Yak SSCs exhibit a regulatory pattern of proliferation-differentiation balance. Transcriptomic data show that in cattle-yak SSCs, the marker genes such as *PAX7*, *ZBTB16*, and *RET*, as well as self-renewal-related genes including *MYC*, are upregulated, while the expression of the maintenance gene *UTF1* is downregulated [93]. Such expression perturbations originate from hybrid genomic incompatibility. These genes contribute to spermatogonial stem cell fate maintenance. Their abnormal expression in cattle-yak is not a direct outcome of high-altitude adaptation. This abnormal gene expression profile is strongly correlated with functional disorders of cattle-yak SSCs. In yak testicular tissues and undifferentiated spermatogonia, the expression level of *OTUD6A* is lower, suggesting a potential association with spermatogonial status. In vitro overexpression experiments have shown that this gene can enhance cell viability and accelerate cell cycle progression [94]; however, these in vitro functional effects still need to be further validated in yak in vivo models. At present, these findings provide preliminary clues for further exploring the potential role of *OTUD6A* in the regulation of yak SSCs.

Regulation of the yak SSC niche shows pronounced differences relative to closely related bovine species. Comparative studies have revealed that the in vitro proliferation capacity and viability of yak SCs are superior to those of cattle-yak; however, the expression level of the *CXCL12* gene in cattle-yak SCs was found to be 3.6-fold higher [92]. This aberrant upregulation occurs against the background of genetic conflict upon hybridization, suggesting the complexity of microenvironmental regulation: elevated expression of a single factor does not equal overall improvement in microenvironment quality. This may reflect compensatory anomalies in cattle-yak SCs induced by genomic incompatibility or disrupted synergistic regulatory networks between *CXCL12* and other microenvironmental factors. These findings indicate that yak SCs may construct a suitable microenvironment for SSCs via the synergistic expression profile of multiple factors, rather than through the difference in a single molecule. However, the specific mechanisms by which high-altitude environments affect the function of yak SSCs still require further in-depth exploration.

### 4.2. Regulation of Meiosis

Meiosis is a critical step in spermatogenesis, and its initiation is tightly regulated by retinoic acid signaling and *STRA8* [95]. Yak spermatocytes sequentially progress through the leptotene, zygotene, pachytene, diplotene, and diakinesis stages to complete homologous chromosome pairing and DNA recombination repair [53,96]. During the pachytene stage, the X and Y chromosomes form a “sex body”, initiating meiotic sex chromosome inactivation (MSCI) [53,97]. Under long-term high-altitude domestication, spermatocyte meiosis in yaks exhibits notable molecular signatures and regulatory mechanisms. The meiotic process of yak spermatocytes exhibits molecular characteristics and regulatory mechanisms, and comparative studies with sterile hybrid offspring provide important clues for elucidating the molecular basis of spermatogenesis arrest. Comparative studies have revealed that spermatocytes of cattle-yaks undergo developmental arrest from the diplotene to diakinesis stage, which is closely associated with defective DNA repair and impaired MSCI [53,98]. (Data from an unpublished dissertation without formal peer review.) Single-cell transcriptomics has identified ten spermatocyte subtypes in the yak testis, revealing a fine-grained molecular map of meiosis [53].

Synaptonemal complex and homologous recombination repair. Among the core meiotic events, synaptonemal complex formation and homologous recombination repair are particularly important. All gene expression abnormalities described for cattle-yak stem from interspecific genetic conflict. These data help identify genes essential for spermatogenesis. They do not reflect high-altitude adaptive traits of yaks. The expression of synaptonemal complex-related genes (e.g., *SYCP1*, *SYCP2*), meiotic recombination genes (e.g., *TEX11*, *MEIOB*), and meiotic progression regulatory genes (e.g., *MSH4*, *STAG3*) is nearly completely lost in cattle-yaks [93]. *SPATA22* is essential for homologous chromosome pairing and DNA damage repair. Its expression is markedly reduced in cattle-yak, and this aberration correlates strongly with meiotic arrest and germ cell loss in hybrid testes [99]. The *SPATA22/MEIOB* complex likewise serves a pivotal function in DNA repair [99]. The specific functions and regulatory mechanisms of this gene in yak GCs still await further experimental validation.

The special status of the *SLX4* endonuclease. Multi-omics integrative analysis revealed that *SLX4* is highly expressed in yak pachytene spermatocytes, downregulated in cattle-yak spermatocytes, but restored to parental levels in backcross progeny [100]. These results suggest that *SLX4* may play a critical role in ensuring proper completion of meiotic homologous recombination, although its causal function awaits in vivo validation. Downregulated *SLX4* expression in cattle-yaks results from genetic conflict upon hybridization, rather than representing high-altitude adaptive variation in yaks. Further studies have revealed that defective expression of this gene in pachytene spermatocytes of cattle-yaks may play a critical role in generating obstacles during homologous recombination, thereby triggering meiotic failure [100]. However, whether its downregulation directly leads to recombination obstruction and meiotic failure still needs to be verified through functional experiments in yak models.

Signaling pathways and cell cycle regulation. The ERK signaling pathway is also involved in the regulation of meiosis. The expression levels of *ERK1* and *ERK2* are higher in yak testes than in cattle-yaks, suggesting that these two genes are required for normal spermatogenesis [101]. Their downregulation in cattle-yaks stems from genetic conflict upon hybridization, and this comparison cannot confirm the involvement of this pathway in high-altitude adaptation. Differential expression of cell-cycle regulatory genes also merits attention. *CDK1* and *CDC25A* play critical roles during meiosis, and their reduced expression levels may be linked to male infertility [102]. *ESCO2* exhibits dynamic expression patterns across different developmental stages of yak testes, with higher expression levels in adulthood than in juvenile and fetal stages. Immunohistochemical results for cattle-yaks demonstrate the absence of ESCO2 protein expression in primary spermatocytes, and this molecular abnormality may be associated with meiotic arrest [103]. The above comparisons between yaks and cattle-yaks attribute their phenotypic differences to the context of hybrid sterility rather than directly reflecting mechanisms underlying high-altitude adaptation.

### 4.3. Spermiogenesis and Sperm Morphogenesis

Spermiogenesis represents the terminal phase of spermatogenesis, encompassing a series of precise morphological differentiation events including acrosome biogenesis, chromatin condensation (protamine replacement), flagellar assembly, and cytoplasmic removal [53,64]. In yaks reared under high-altitude conditions, the first wave of spermatozoa can be observed in testicular tissue at 12 months of age, marking the cytological initiation of spermatogenesis. Full-scale expression of LDH-X occurs at 24 months of age as a hallmark of maturation, indicating that the testes have attained molecular-physiological functional maturity with the complete establishment of spermatogenesis-related metabolic pathways [64]. However, cytological and molecular maturation do not equate to reproductive readiness for breeding individuals. Constrained by hypoxia and limited energy availability, yaks require additional time for full maturation of HPG axis function, physical condition, and semen stability. Single-cell transcriptomics has identified 11 spermatid subtypes in the yak testis, revealing the state transition trajectories during spermiogenesis [53]. The epididymis, serving as the site of sperm maturation, mediates sperm membrane modification and motility acquisition through the secretion of proteins and androgens [104].

Abnormal flagellar development represents a prominent manifestation of sperm morphological defects in cattle-yaks, which is caused by hybrid genomic incompatibility. Genomic analysis revealed that the expression levels of several key genes involved in ciliogenesis and flagellar assembly were downregulated in cattle-yaks, suggesting that these genes are indispensable for sperm morphogenesis [93]. Aberrant regulation of acrosome formation and protamine replacement. The current understanding of the molecular mechanisms underlying acrosome formation in yaks remains incomplete. However, based on the hybrid-defective phenotype of abnormal sperm acrosome morphology in cattle-yaks, it is reasonable to speculate that genes related to acrosome development may likewise exhibit expression defects. Nevertheless, the above-mentioned speculations fall within the scope of the fundamental biology of spermatogenesis and cannot be extrapolated to high-altitude adaptive evolution. Comparative transcriptomic analysis revealed a downregulation of multi-gene clusters in cattle-yak associated with cell morphogenesis, spermatogenesis, acrosome formation, ciliogenesis, and flagellar development [93]. Such dysregulated co-expression of multiple genes may play a vital role in impairing the structural integrity of the sperm acrosome, thereby disturbing the normal progression of sperm-oocyte recognition and fertilization. Taken together, all the above-described abnormalities represent molecular phenotypes accompanying hybrid sterility and should not be directly regarded as characteristics of high-altitude stress responses.

Recent studies have shown that differential expression of the *SPATA3* gene affects the sperm maturation process [105], and further research has revealed that *SPATA3* may participate in clearing redundant organelles and protein components generated during sperm cell differentiation by regulating the apoptosis pathway and the autophagy degradation system, thereby ensuring normal sperm cell development. Notably, spermatogenesis in cattle-yaks is arrested at meiosis owing to hybridization, and spermiogenesis fails to initiate. Nevertheless, a small number of elongated spermatozoa present in first-generation backcross progeny can support embryonic development following intracytoplasmic sperm injection (ICSI), which confirms the decisive role of intact spermiogenesis for fertilization competence [53].

The various stages of spermatogenesis are not only precisely regulated by the intrinsic programs of GCs but also highly dependent on the structural support, nutritional supply, and signaling cues provided by the cells of the testicular microenvironment. Therefore, elucidating the regulatory roles of microenvironmental cells in spermatogenesis is indispensable for a comprehensive understanding of the molecular mechanisms underlying spermatogenesis in yaks.

## 5. Regulation of Spermatogenesis by the Testicular Microenvironment

Testicular niche cells play a pivotal role in directing the survival and differentiation of male GCs, and spermatogenesis relies on the full maturation of the somatic microenvironment [106]. In the adult testis, niche cells, including SCs, LCs, and myocytes (MCs), provide a microenvironment that enables SSCs to successfully complete spermatogenesis [107]. Therefore, mammalian spermatogenesis is a complex process that requires intricate interactions between somatic cells and GCs [108].

### 5.1. Sertoli Cells

SCs are the “nurse cells” of the seminiferous tubules and the only somatic cell type that directly contacts the GCs within them [109]. During mammalian embryonic development, mesenchymal progenitor cells differentiate into SCs, which produce AMH to inhibit the development of the female internal reproductive tract [86]. During the neonatal stage, SCs direct the migration of GCs toward the basement membrane of the seminiferous tubules and stimulate their proliferation to establish the spermatogonial SSC pool [110]. SCs also assemble the BTB through desmosomes, gap junctions, and tight junctions, which segregate the seminiferous tubule lumen from the basal compartment. Meanwhile, SCs secrete multiple cytokines such as transforming growth factor-β (TGF-β), tumor necrosis factor-α (TNF-α), interleukin-1α (IL-1α), and interleukin-6 (IL-6). These molecules build a local cytokine network within the testis to modulate testicular immune function and sustain its immune-privileged status. In addition, SCs provide physical support, nutrients, and a variety of growth factors for spermatogenic cells [111], including secretion of several critical regulatory factors such as GDNF, stem cell factor (SCF), androgen-binding protein (ABP), bone morphogenetic protein 4 (BMP4), inhibin-β, insulin-like growth factor-1 (IGF-1), chemokines (*CXCL12*), and FGF2. Among these factors, GDNF and FGF2 synergistically regulate *CXCR4* expression in SSCs, thereby modulating the *CXCL12-CXCR4* signaling pathway to participate in self-renewal and proliferation of SSCs. Additionally, SCs phagocytose apoptotic GCs and their residual bodies to maintain testicular homeostasis [112,113]. SCs exert multiple core regulatory functions during testicular organ development and spermatogenesis.

### 5.2. Leydig Cells and Myocytes

LCs and MCs share a common progenitor before puberty [114]. The hypothalamus releases GnRH, which acts on the pituitary to stimulate the anterior pituitary to secrete LH and FSH, among which LH can stimulate mesenchymal cells to differentiate into LCs, promoting the synthesis and secretion of testosterone [115,116]. Testosterone can act on MCs, LCs, and SCs via the androgen receptor [117]. Additionally, LCs can secrete IGF1 and CSF1 to regulate spermatogenesis [118]. Peritubular myoid cells (PMCs) surrounding seminiferous tubules provide structural support and produce peristaltic contractions that facilitate sperm transport out of tubules. Additionally, PMCs exhibit secretory capabilities, producing a variety of bioactive substances to modulate the functions of LCs and SCs [119]. Consequently, SCs, LCs, and PMCs constitute an intricate communication network, establishing a tightly regulated microenvironment for GCs to modulate spermatogenesis [120].

## 6. Epigenetic Regulatory Mechanisms

Beyond intercellular communication within the microenvironment, spermatogenesis is subject to exquisite epigenetic regulation. The epigenetic regulation of spermatogenesis is orchestrated by the synergistic action of DNA methylation, histone modifications, and non-coding RNAs, involving dynamic chromatin remodeling and specific reprogramming of methylation patterns. Environmental factors, such as nutrition and metabolic abnormalities, can induce plastic changes in the sperm epigenome, which may be transmitted across generations via epigenetic memory. High-throughput sequencing technologies now enable the integration of three-dimensional chromatin conformation data to investigate the spatiotemporal reorganization patterns of topologically associating domains (TADs) during spermatogenesis, providing new insights into the reproductive adaptation mechanisms of high-altitude species.

### 6.1. DNA Methylation Reprogramming

During spermatogenesis, DNA methylation undergoes complex dynamic changes. In mice, global genomic methylation levels progressively decline from meiosis to elongated spermatid differentiation [121]. During this process, somatic methylation patterns are erased, providing a prerequisite for the subsequent establishment of parent-specific (parental-specific) methylation patterns. In cattle-yaks, driven by genomic conflict arising from interspecific hybridization, SCs exhibit prominent differences in DNA methylation modifications [122]. Whole-genome analyses reveal that testicular methylation levels in cattle-yaks are higher than in purebred yaks, accompanied by upregulated *DNMT3A* expression [123]. Meanwhile, multiple key genes of the PIWI/piRNAs pathway exhibit promoter hypermethylation; this observation correlates with diminished piRNAs production during the pachytene stage, suggesting that *DNMT3A* upregulation may contribute to the above-mentioned epigenetic abnormalities, although their direct regulatory relationship remains to be further validated [124]. The aforementioned methylation abnormalities also represent molecular correlates of hybrid sterility rather than epigenetic markers for plateau adaptation. However, genome-wide methylation studies in yak testes remain insufficient, and most mechanistic insights are extrapolated from mouse or human models, requiring yak-specific validation.

### 6.2. Histone Modifications

At the level of histone modifications, histone acetylation (e.g., *H3K27ac*, *H4ac*) and methylation (e.g., *H3K4me3*, *H3K9me3*) regulate chromatin accessibility and gene transcriptional activity during mammalian spermatogenesis, serving as a bridge connecting external environmental signals to gene expression programs [125,126]. In model animals such as mice, histone deacetylases (HDACs) and histone methyltransferases (e.g., *PRDM9*) play critical roles in meiotic recombination and spermatocyte differentiation. Their dysregulated expression is associated with aberrant synaptonemal complex formation and meiotic arrest [127,128]. In yaks, research on histone modifications remains very limited; now, only the expression changes in some modifying enzymes (such as the *KDM* family and *HDAC* family) have been inferred from the transcriptome level, and direct functional evidence remains lacking. There is an urgent need to map the dynamic landscape of histone modifications during yak spermatogenesis through techniques such as ChIP-seq and CUT&Tag, thereby revealing how high-altitude environmental factors regulate reproductive gene expression through epigenetic modifications.

### 6.3. Regulation by Non-Coding RNAs

Non-coding RNAs (nc-RNAs) refer to transcripts that do not encode proteins but perform biological functions in the form of RNA molecules [129]. Transcriptomic studies have demonstrated that only approximately 2% of bases in the mammalian genome encode proteins, whereas the vast majority of remaining transcripts are nc-RNAs whose functions are being progressively elucidated [130]. Nc-RNAs serve as regulators of gene expression and are extensively involved in key biological processes such as cell proliferation, differentiation, apoptosis, and reproduction [129]. Based on transcript length, ncRNAs can be classified into two major categories: small nc-RNAs (less than 200 nt) and long ncRNAs (lncRNAs, greater than 200 nt). Small nc-RNAs mainly include microRNAs (miRNAs), PIWI-interacting RNAs (piRNAs), small interfering RNAs (siRNAs), and small nucleolar RNAs (snoRNAs) [131]. Deep sequencing identified 737 known and 359 novel miRNAs in yak testes with stage-specific expression during development, notably revealing that bta-miR-2290 influences testicular development and spermatogenesis by regulating the MAPK signaling pathway [42].

PiRNAs in yak testes exhibit diverse genomic origins, with 13.44% derived from intergenic repetitive sequences, 21.40% from genic regions, and 65.15% mapping to other regions [28]. PiRNAs play a role during meiosis through epigenetic modification and post-transcriptional gene silencing [28]. PIWI proteins synergize with piRNAs to mediate mRNA decay in SPTs, whereas the APC/C ubiquitination pathway participates in protein degradation. LncRNAs exert regulatory functions through multiple mechanisms: at the epigenetic level, they participate in DNA methylation, chromatin remodeling, and histone modifications; at the transcriptional level, they act as enhancer RNAs to facilitate enhancer-promoter looping and gene activation; and at the post-transcriptional level, they regulate mRNAs stability and translation via base-pairing complementarity.

**Figure 3:**
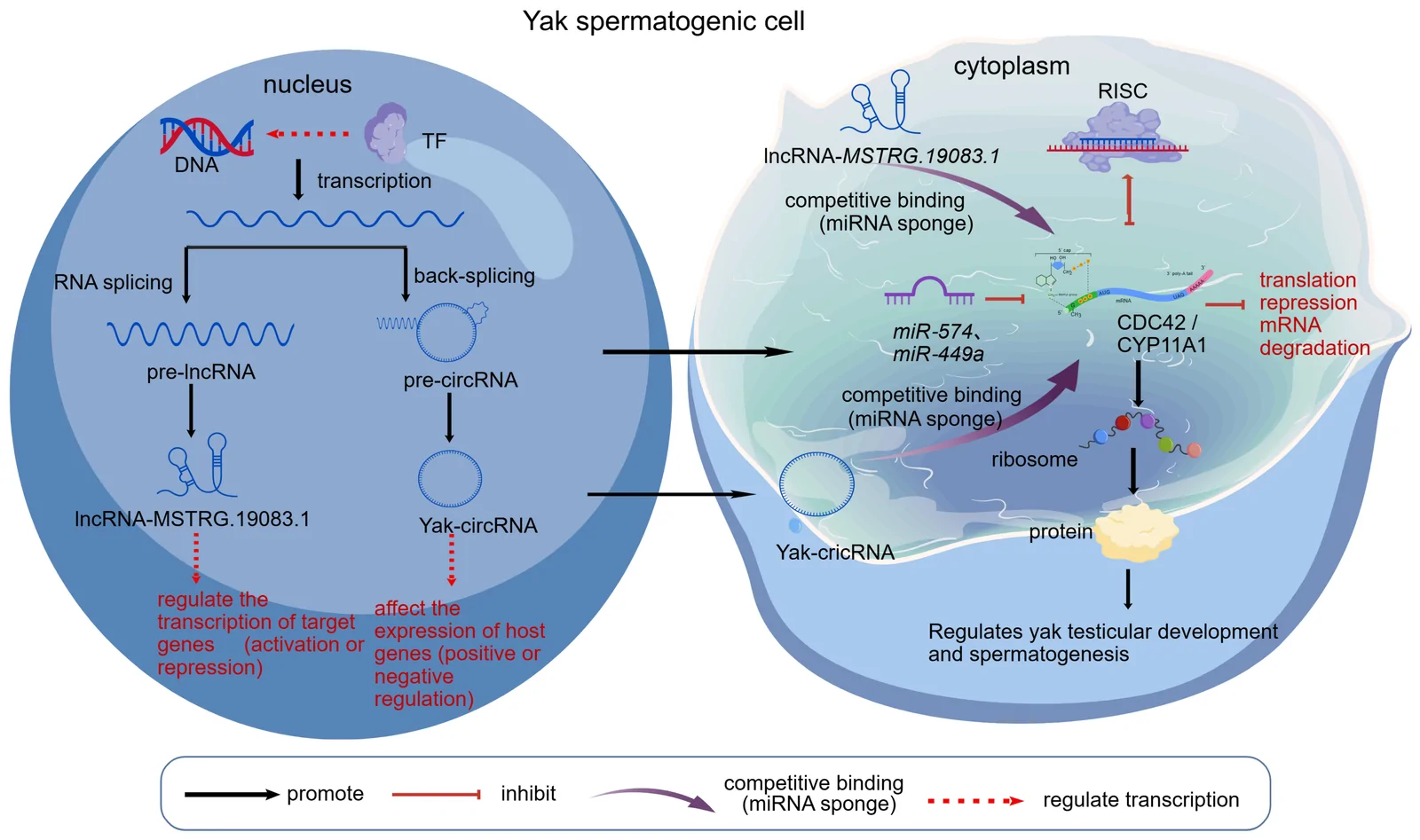
Schematic diagram of ceRNA regulatory mechanisms during yak testicular development. This illustration depicts the ceRNA regulatory network composed of lncRNAs-circRNAs-miRNAs-mRNAs in yak spermatogenic cells, which participates in testicular development and spermatogenesis in yaks. The ceRNA sponge network presented in this diagram is a conceptual model inferred from transcriptomic correlation analyses; definitive functional validation in yak germ cells is still lacking.

Non-coding RNAs exert regulatory functions synergistically through complex interaction networks. The competing endogenous RNAs (ceRNAs) mechanism represents one of the most thoroughly investigated regulatory paradigms [132]. The core of this mechanism is that lncRNAs and circRNAs can act as miRNA sponges. By competitively sequestering available miRNA molecules, they de-repress target mRNAs, thereby forming a “lncRNAs/circRNAs-miRNAs-mRNAs” regulatory axis. In the Nanos2 knockout mouse model, researchers constructed a ceRNAs regulatory network comprising miRNAs, circRNAs, lncRNAs, and mRNAs, revealing the interactions between coding and nc-RNAs in regulating SSC generation [132]. In the testes of post-pubertal Holstein bulls, a circRNAs-miRNAs-mRNAs regulatory network has also been identified, suggesting that circRNAs may be involved in the regulation of spermatogenesis in the adult testis. However, this conclusion is primarily based on transcriptomic association analyses and network predictions, and the direct regulatory functions and causal relationships still need to be further validated through in vivo functional experiments [133]. In studies on yak testicular development, whole-transcriptome sequencing analysis has preliminarily constructed a circRNAs-miRNAs-mRNAs regulatory network, revealing that differentially expressed ncRNAs, such as miR-574 and miR-449a, may be involved in testicular spermatogenesis (Figure 3) [5]. Furthermore, some lncRNAs can serve as precursors for piRNAs, indirectly participating in piRNA-mediated transposon silencing and the maintenance of genomic stability, further highlighting the functional synergy among different types of nc-RNAs.

## 7. Population Genetics and Tissue Expression Profiling of the *EPAS1/HIF* Pathway

Owing to long-term habitation of yaks on the Qinghai–Tibet Plateau at altitudes of 4000–5000 m, the mechanisms underlying hypoxia adaptation have become an important research direction in evolutionary genetics. The *EPAS1*/*HIF* signaling pathway plays a central role in the regulatory network for hypoxia adaptation in yaks [134]. Endothelial PAS domain-containing protein 1 (*EPAS1*) encodes hypoxia-inducible factor-2α (HIF-2α). Together with HIF-1α, it belongs to the HIF transcription factor family and acts as a core molecule regulating cellular oxygen sensing and hypoxia responses [135]. In the adaptive evolution of plateau domestic animals, *EPAS1* represents a major gene under pervasive positive selection in plateau species, including yaks and Tibetan sheep. It mediates organismal adaptive survival under plateau hypoxia mainly by regulating pathways such as erythropoiesis and angiogenesis [136].

Population genetic studies have identified three highly linked species-specific SNP loci within the yak *EPAS1* gene, namely g.83052C>T, g.83065G>A, and g.83067C>A [137]. Using the allelic combinations of these three tightly linked SNPs, the researchers genotyped yak individuals and defined several genotypes (AA, AB, AC, BC, and CD), with the CD genotype being the primary focus. Population analyses show that the CD genotype of *EPAS1* is enriched in yak populations inhabiting altitudes above 3000 m. Individuals carrying this genotype exhibit markedly higher hemoglobin concentrations than those with other genotypes. Neutrality tests further confirm that these SNP loci are under directional selection imposed by the plateau hypoxic environment, verifying *EPAS1* as an important molecular marker for hypoxia adaptation in yaks [137]. Tissue expression profiles from non-reproductive organs show that *EPAS1* is broadly expressed in yaks, with the highest transcript abundance in lung tissue, and it is also detectable in the liver, kidney, pancreas and other tissues [137].

Data regarding the expression and function of *EPAS1* in yak testicular tissue remain largely unavailable. Nevertheless, studies using mouse models have confirmed that *EPAS1* exerts conserved and critical biological functions in mammalian testicular development and spermatogenesis, which can provide important references for related research on yaks [138]. Combining the population genetic signatures of positive selection on *EPAS1* under plateau hypoxia in yaks and the conserved functions of this gene in mammals, it can be speculated that the yak testis, a naturally hypoxic organ, may rely on *EPAS1*-mediated hypoxia-response pathways to maintain the homeostasis of the spermatogenic microenvironment, thereby guaranteeing normal progression of spermatogenesis following long-term residence in hypoxic habitats. Nevertheless, this speculation currently lacks direct experimental evidence concerning the expression patterns and cellular localization of *EPAS1* in yak testicular tissues.

## 8. Advances in the Application of Technical Methods

The rapid advancements in molecular biology, particularly the innovative applications of single-cell omics, gene editing, and bioinformatics, have provided powerful tools to unravel the reproductive regulatory mechanisms of yaks in high-altitude environments. This is driving the field to pivot from phenotypic descriptions to mechanistic investigations.

### 8.1. Integrative Multi-Omics Analysis

Single-cell transcriptomic analysis elucidates the heterogeneity and developmental trajectory of yak GCs. Using this technology, the research team has defined the testicular cell transcriptomic profiles of cattle, yaks and cattle-yaks. With the hybrid sterility model, they further uncovered the molecular mechanisms underlying abnormal spermatogonial cell fate determination and meiotic initiation driven by genomic incompatibility in cattle-yaks [53,139].

Integrated multi-omics analysis (including ATAC-seq, mRNA-seq, and proteomics) revealed the correlation between chromatin accessibility and gene expression and identified core regulatory genes [140]. However, with as many as 11,904 differentially expressed transcripts arising during the gonocyte-to-spermatogonia transition, data integration and functional analysis present substantial challenges, and the co-regulatory networks linking non-coding RNAs and coding RNAs remain to be explored [4,141].

### 8.2. Gene Editing and Functional Validation

CRISPR/Cas9 technology has shown great value in studying interspecific hybrid sterility, as demonstrated by the finding that functional repair of the *SPATA22* gene can enhance the fertility of hybrid offspring [99]. Nevertheless, the poor transfection efficiency of primary cells poses a major bottleneck. On the other hand, testicular organoid models can mimic the in vivo microenvironment. Firstly, they can be applied to construct controllable in vitro systems for investigating the effects of plateau hypoxia stress on testicular development in purebred yaks. Secondly, they can act as in vitro assay systems for CRISPR-edited constructs and reduce reliance on animal experiments. Integrating CRISPR with single-cell transcriptomics enables the evaluation of gene editing effects at single-cell resolution, representing a key direction for building a yak-specific functional validation platform.

### 8.3. Bioinformatics Analysis Tools

Bioinformatics tools serve as a bridge for extracting biological insights from massive omics datasets. Depending on the depth of analysis, these tools can be categorized into data mining, functional annotation, and structural prediction. At the data mining level, WGCNA was utilized to construct a co-expression network, identifying 21 modules and core hub genes associated with developmental stages of yak testes [67]. Through the combined screening of multiple software tools such as CNCI and CPC2, a total of 5130 lncRNAs were identified from the transcriptomic data, among which 1367 were differentially expressed [142]. At the functional annotation level, GO and KEGG enrichment analyses revealed the functional characteristics of differentially expressed proteins and the aberrant metabolic pathways [32]. At the structural prediction level, tools such as ProtParam and SWISS-MODEL were utilized to predict the physicochemical properties and three-dimensional structures of proteins like SIRT3 and SPATA3 [66,105]. Furthermore, integrative analysis of genomic structural variations and transcriptomic data identified 661 reactivated genes harboring structural variants in backcross progeny [53]. Combined with spermatocyte proteomic data, 24 candidate genes were screened out, which provide critical clues for uncovering the mechanism of hybrid sterility [53].

## 9. Sperm Cryopreservation and Fertility Assessment in Yaks

### 9.1. Yak Sperm Cryopreservation

Sperm cryopreservation is a core biotechnology for conserving yak germplasm and genetic resources, promoting artificial insemination and propagation of elite breeding bulls. It also provides material support for cattle-yak crossbreeding and the implementation of assisted reproductive technologies [143]. Although yak sperm have long adapted to high-altitude conditions of hypoxia and strong ultraviolet radiation, these animals have evolved distinct energy metabolism and oxidative stress profiles in their gametes. Nevertheless, they are extremely susceptible to osmotic fluctuations and oxidative damage induced by freeze-thawing [144]. Data summarized by Kalwar et al. [145] showed that post-thaw motility of yak sperm reached 50.55% when a 12% sucrose-based extender was used, whereas skim-milk- or sodium-citrate-based extenders yielded only approximately 30.4%. This indicates that directly adopting extenders optimized for ordinary cattle significantly impairs post-thaw sperm quality in yaks.

Current research on yak semen cryopreservation mainly focuses on the modification of extender formulations and screening of exogenous protective additives. The glycerol egg-yolk-Tris-based extender represents a classic system for yak semen cryopreservation; the final glycerol concentration and equilibration time in this system significantly affect post-thaw sperm recovery [146,147]. Numerous studies have verified that adding antioxidant components, including DHA [148], ginseng polypeptides [149] and artesunate [150] to cryo extenders improves post-thaw total sperm motility, progressive motility, plasma membrane and acrosome integrity, decreases malondialdehyde accumulation and elevates in vitro fertilization rates by scavenging reactive oxygen species, boosting antioxidant enzyme activities and suppressing the mitochondrial apoptotic pathway.

As a seasonally breeding species, yaks exhibit marked seasonal variations in semen quality, and collection timing directly influences cryopreservation outcomes [151]. Accordingly, semen samples from the breeding season should be prioritized in semen cryopreservation experiments and on-farm practice, with confounding variables including age and rearing environment controlled to eliminate seasonal-plasticity-derived interference. These seasonal differences essentially represent sperm molecular remodeling at transcriptomic and proteomic levels, thereby altering tolerance to cryostress. Nevertheless, molecular mechanisms responsible for yak sperm cryodamage are still poorly elucidated, and further research is needed for developing dedicated protective systems and standardized detection of damage biomarkers.

### 9.2. Fertility Assessment Parameters of Breeding Bulls

Accurate fertility assessment for yak breeding bulls serves as an essential prerequisite for superior breed selection and industrial efficiency gains. Traditional selection primarily depends on the bulls’ own phenotypic records and progeny testing [152]. Nowadays, such assessments also incorporate reproductive traits, including testicular volume and scrotal circumference, as well as semen parameters such as ejaculate volume, sperm concentration, sperm morphological abnormality rate and post-thaw recovery rate. Nevertheless, reliance solely on these indicators is insufficient to predict the actual reproductive capacity of breeding bulls.

With the rapid advancement of omics technologies, potential molecular markers for fertility in domestic animals have been continuously identified and validated. Among them, sperm-derived miRNAs can effectively discriminate yak breeding bulls with high fertility from those with subfertility [37]. Hypotheses also suggest that the expression levels of heat-shock proteins and seminal plasma protein profiles may correlate with yak sperm quality, with potential utility as auxiliary biomarkers for fertility evaluation; nevertheless, this hypothesis awaits further validation. Within cross-breeding systems, fertility assessment also includes the detection of spermatogenesis restoration levels in male backcross progeny. Inspired by cattle-yak male sterility, backcross bulls require semen phenotypic examination and molecular screening to determine whether the arrest of spermatogenesis has been relieved [53].

Now, the comprehensive evaluation system for yak breeding bulls adapted to high-altitude farming environments remains imperfect, and the synergistic application of morphological phenotypes, molecular markers and on-farm conception data has not yet been realized. Establishing a robust, comprehensive fertility assessment system can not only provide a basis for the precise selection of high-quality breeding bulls but also lay a practical foundation for breaking the existing bottlenecks in cattle-yak cross-breeding.

## 10. Research Perspectives

The present review summarizes research progress regarding the molecular regulation of testicular development and spermatogenesis in yaks. Nevertheless, current omics-based comparative studies between yaks and low-altitude ordinary cattle suffer from multiple confounding interferences. These two species differ not only in altitude-related environmental conditions but also in variables such as genetic background, breeding season and rearing conditions. Yaks are typical seasonal breeding species. However, most comparative sampling designs have not strictly controlled for sampling seasons, so observed differences in testicular gene expression may originate from seasonal physiological fluctuations. They may not fully represent genetic adaptive variations generated by long-term high-altitude selection. Complete matching of all covariates is difficult under field-sampling conditions, which complicates causal inference. Future experimental designs should unify factors including season, age and rearing conditions as far as possible and combine time-series sampling with in vitro models to effectively discriminate the effects of seasonal plasticity from those of genetic adaptation.

With the rapid advancement of omics and analytical technologies, significant progress has been made in elucidating the molecular regulatory mechanisms of yak testicular development and spermatogenesis. However, compared with model species such as humans, mice, and cattle, research on yaks is still in its infancy. Future studies should integrate multi-omics technologies to construct a comprehensive molecular regulatory network spanning the entire lifespan, from embryogenesis and adulthood to aging. Specifically, it is crucial to elucidate the spatiotemporal differentiation trajectories and interaction networks of various somatic cells (e.g., SCs and LCs) and spermatogenic cells, thereby revealing the regulatory effects of genomic structural variations on testicular development and spermatogenesis.

At the level of data analysis and result interpretation, two independent biological events should be strictly distinguished: spermatogenic defects triggered by interspecific genomic incompatibility in cattle-yaks and the genetic and expression differences between purebred yaks and plain cattle, as well as among yaks from different altitudes. Genes identified using the cattle-yak sterility model are essential for core spermatogenesis. These genes cannot be directly interpreted as candidates for high-altitude reproductive adaptation in yaks. Embryonic gonad samples from yaks remain extremely difficult to acquire. Most conclusions about early developmental events, including the *SRY-SOX9* sex-determination cascade and primordial gonocyte migration and colonization, are inferred from conserved pathways in model animals. Direct in situ experimental evidence from yak tissues is scarce. Future studies should focus on overcoming the technical obstacles in embryonic gonad sample collection to acquire direct experimental data covering the gonadal differentiation stage of yaks.

High-altitude hypoxia represents the most pivotal environmental stressor shaping the male reproductive phenotype of yaks; nevertheless, direct molecular evidence at the testicular tissue level remains very scarce. *EPAS1* is a core hypoxia-responsive gene under positive high-altitude selection in yaks. However, its cellular localization, temporal expression profiles and biological functions in yak testes remain unclear. Further studies can employ techniques such as qPCR and Western blot to characterize *EPAS1* expression across distinct testicular cell types and developmental stages. Such work will help clarify the molecular pathways through which the *EPAS1*/*HIF* axis contributes to testicular development, spermatogenesis, and high-altitude reproductive adaptation.

On this basis, it is critical to promote the translation of fundamental research into practical production applications. Further efforts should be made to explore male fertility molecular markers applicable to breeding bull selection and establish an integrated fertility evaluation system that combines morphological phenotypes, molecular markers, and on-farm conception rates. Moreover, elucidating the molecular mechanisms underlying yak sperm cryodamage under high-altitude conditions can facilitate the iterative optimization of semen cryoprotection systems. Collectively, these studies will provide theoretical support for breaking the bottleneck of male sterility in cattle-yaks and improving the crossbreeding system of plateau yaks.

## 11. Conclusions

Yaks inhabit the extreme high-altitude environment of the Qinghai–Tibet Plateau characterized by hypoxia and large diurnal temperature fluctuations and have evolved distinct adaptive features in male reproduction. Testicular weight is significantly lower than that of yellow cattle. Summary data of semen parameters from different studies indicate that although ejaculate volume is somewhat reduced, sperm motility ranges overlap with those of yellow cattle, suggesting that yaks may possess higher spermatogenic efficiency per unit of testicular tissue. However, this inference remains to be validated by comparative studies under rigorously controlled and uniform experimental conditions. Specialized counter-current heat-exchange vessels within the spermatic cord establish a low-temperature microenvironment for the testes. Meanwhile, molecules such as heat-shock proteins are highly expressed in testicular tissues, which collectively maintain normal spermatogenesis under high-altitude stress. This review summarizes the complete developmental trajectory of yak testes, ranging from embryonic gonadal differentiation, structural establishment during the juvenile stage, activation of the HPG axis at puberty, functional homeostasis in adulthood, to degenerative senescence in old age. Notably, the mechanisms underlying embryonic-stage events are largely inferred from conserved signaling pathways in model animals. It further dissects the molecular regulatory networks underlying SSCs’ renewal and differentiation, meiosis, and spermiogenesis. It characterizes the testicular microenvironment formed by SCs and LCs, as well as the functions of epigenetic modulators such as DNA methylation, histone modification, and nc-RNAs in reproductive development. Taking cattle-yak hybrid male sterility as a natural research model, a panel of conserved essential genes for spermatogenesis is identified. It is also emphasized that these genes only represent basal regulatory elements of spermatogenesis and should not be directly considered as high-altitude reproductive adaptive genes in yaks. However, several critical gaps remain in this field. Constrained by the limited accessibility of yak embryonic samples, direct experimental evidence for gonadal differentiation in yak embryos is severely lacking. Most inferences regarding early gonadal development are drawn from conserved mechanisms in model organisms such as mice, and in vivo validation in yak tissues is still absent. Current studies on high-altitude reproductive adaptation are mostly based on omics comparisons between yaks and low-land cattle. Confounded by factors such as species evolutionary background and breeding season, covariates cannot be fully controlled in field sampling. Some molecular differences may stem from environmental plasticity or seasonal physiological fluctuations and thus should not be simply attributed to genetic adaptive evolution induced by high altitude. In addition, existing epigenetic studies are largely restricted to transcriptomic profiling analyses, with a scarcity of high-quality epigenomic datasets. Most candidate genes are only supported by omics-derived correlative evidence, and in vivo and in vitro functional validations remain lacking. These aforementioned issues warrant further investigation in future studies.

## Figures and Tables

**Table 1:** Genes and functions relevant to yak embryonic testicular development (evidence mainly from model animals).

Gene	Related Function	Cell Type	Species (Data Source)	Developmental Stage	References
*SRY*, *SOX9*	Sex determination, differentiation of genital ridge toward testis	Coelomic epithelium of genital ridge	Cattle, mouse	Embryonic gonadal development	[7,12,13]
*NR2F2*, *HGF*, *c-met*	Differentiation of fetal interstitial cells	Fetal interstitial cells	Mouse	Embryonic gonadal development	[15,16]
*GATA4*, *AMH*	Sertoli cell differentiation, Müllerian duct regression	Sertoli cells	Mouse	Embryonic gonadal development	[17]
*CXCR4*, *CXCL12*, *TCF21*, *ACTA2*	PGC migration and microenvironment (niche) establishment	Primordial germ cells, interstitial cells	Mouse, macaque	Embryonic gonadal development	[16,18,19]
*NANOG*	Maintenance of PGC survival	Primordial germ cells	Mouse	Embryonic gonadal development	[20]
*DDX4*	Regulation of PGC proliferation	Primordial germ cells	Mouse	Embryonic gonadal development	[21]
*TCFAP2C*	Suppression of somatic cell differentiation program	Primordial germ cells	Mouse	Embryonic gonadal development	[22]
*STAR*	Cholesterol transport, initiation of steroidogenesis	Leydig (interstitial) cells	Mouse	Embryonic gonadal development	[23]
*HSD3B2*, *CYP17A1*	Key enzymes in androgen synthesis	Leydig cells	Human	Embryonic gonadal development	[24,25]
*PIWIL4 (MIWI2)*	Mediation of transposon silencing, genomic methylation	Germ cells	Yak, Mouse	Embryonic–juvenile testis	[26,27,28]
*MSL3*	Epigenetic regulation	Germ cells	Primates, rodents	Embryonic gonadal development	[18,29]

**Table 2:** Comparison of testicular and semen parameters between yaks and cattle. Data in this table are compiled from independent studies conducted at different sampling sites (with varying altitudes) and across different seasons, representing a summary of literature findings rather than results from direct paired comparisons.

Parameter	Yak	Cattle	Remarks	References
Average weight of unilateral testis (g)	95.80 ± 26.27	278.53 ± 56.50	Adult slaughtered individuals; measured with a vernier caliper and electronic balance; sampling site: Xining, Qinghai (altitude approx. 2260 m)	[77]
Total length of epididymis (mm)	165.00 ± 21.45	221.83 ± 15.85	Adult slaughtered individuals; measured with flexible tape	[77]
Semen volume (mL)	3–4	4.80 ± 1.60	Yak: semen collected from July to October, adult breeding bulls, artificial vagina; Tibetan cattle: sampled in summer in Tibetan area, artificial vagina; data from different experiments	[78,79]
Fresh sperm motility (%)	67–71	55.64–70	Microscopic examination (400×); yak semen collected from July to October	[78,79]
Sperm concentration (×10^8^/mL)	11.00–15.00	12–15.74	Counted with a hemocytometer; yak semen collected from July to October	[78,79]

## Data Availability

No new data were created or analyzed in this study. Data sharing is not applicable to this article.

## Abbreviations

The following abbreviations are used in this manuscript:

SCs	Sertoli cells
LCs	Leydig cells
PMCs	Peritubular myoid cells
AMH	Anti-Müllerian hormone
GCs	Germ cells
PGC	Primordial germ cells
HPG	Hypothalamic-pituitary-gonadal
GnRH	Gonadotropin-releasing hormone
GnRHR	Gonadotropin-releasing hormone receptor
FSH	Follicle-stimulating hormone
LH	Luteinizing hormone
FSHR	Follicle-stimulating hormone receptor
LHR	Luteinizing hormone receptor
HIF	Hypoxia-inducible factor
GH	Growth hormone
PRL	Prolactin
PRLR	Prolactin receptor
AR	Androgen receptor
INHβB	Inhibin β-B subunit
Pri-SPCs	Primary spermatocytes
Sec-SPCs	Secondary spermatocytes
SPTs	Spermatids
SSCs	Spermatogonial stem cells
GDNF	Glial cell line-derived neurotrophic factor
MSCI	Meiotic sex chromosome inactivation
MCs	Myocytes
BTB	Blood-testis barrier
TGF-β	Transforming growth factor-β
TNF-α	Tumor necrosis factor-α
IL	Interleukin
SCF	Stem cell factor
ABP	Androgen-binding protein
BMP4	Bone morphogenetic protein 4
ncRNA	Non-coding RNA
miRNAs	MicroRNAs
piRNAs	PIWI-interacting RNAs
siRNAs	Small interfering RNAs
snoRNAs	Small nucleolar RNAs
ceRNA	Competing endogenous RNA
EPAS1	Endothelial PAS domain-containing protein 1
HSPs	Heat shock proteins
